# Resistance Training and Stroke: A Critical Analysis of Different Training Programs

**DOI:** 10.1155/2017/4830265

**Published:** 2017-12-20

**Authors:** Bruno Bavaresco Gambassi, Hélio José Coelho-Junior, Paulo Adriano Schwingel, Fabiano de Jesus Furtado Almeida, Tânia Maria Gaspar Novais, Paula de Lourdes Lauande Oliveira, Bismarck Ascar Sauaia, Cristiane Dominice Melo, Marco Carlos Uchida, Bruno Rodrigues

**Affiliations:** ^1^Faculty of Physical Education, University of Campinas, Campinas, SP, Brazil; ^2^Human Performance Research Laboratory, University of Pernambuco, Petrolina, PE, Brazil; ^3^Physical Education Department, Ceuma University, São Luis, MA, Brazil

## Abstract

The aim of this study was to carry out a literature review on the overall benefits of resistance training (RT) after stroke and undertake a critical analysis of the resistance exercise programs surveyed (rest interval between sets and exercises, number of sets, number of repetitions, intensity, duration of training, and weekly frequency). To obtain articles for the review, we searched PubMed, Google Scholar, and Physiotherapy Evidence Database (PEDro). Inclusion criteria were considered using the PICO (population, intervention, control/comparison, and outcome variables) model. The following characteristics were recorded for all articles: type of study, author, year of publication, participants (time after stroke, sample size, and age), benefits of RT, and structured resistance exercise programs. Positive effects of training were found on anxiety status, quality of life, muscle hypertrophy, cognitive function, strength, and muscle power. Only 5 studies described the main variables of RT in detail. Lack of control of some variables of RT may negatively affect the results of this practice. The findings of the present study may further inform health and physical conditioning professionals on the importance and necessity of using the main variables in the search for benefits for individuals with stroke.

## 1. Introduction

Stroke is generally defined as a neurological loss caused by an abnormal perfusion of brain tissue. The most common types of stroke are intracerebral hemorrhage, subarachnoid hemorrhage, and ischemic (cerebral infarction) [1]. A recent projection from the American Heart Association (AHA) [2] indicated that, by 2030, 3.4 million people aged ≥18 years will have had a clinical diagnosis of stroke. These data deserve concern, once this disease is characterized by a poor prognosis, from—at least—a marked impairment of functional capacity and cardiorespiratory fitness due to central and peripheral mechanisms until early death [3–5]. In fact, in 2013, stroke was the cause of 1 in every 20 deaths in the US, and every 40 seconds someone is affected by this disease [2].

Several risk factors have been suggested to be associated with the physiopathology of stroke, such as hypertension, diabetes mellitus, atrial fibrillation, high triglycerides, tobacco use, inadequate diet, family history and genetics, gender, atherosclerosis, chronic kidney disease, sleep apnea, and physical inactivity [2, 6–16]. Regarding the last one, indeed, not only is physical inactivity known to be associated with the development of some, if not all, of the aforementioned risk factor for stroke, but also data demonstrate that moderately active individuals and highly active individuals have a 20% and 27%, respectively, lower risk stroke incidence than low-active individuals [17].

On the other hand, evidences have demonstrated that exercise training has a strong capacity to collaborate with changes on cardiorespiratory capacity, mobility, cognition, upper and lower limb motor capacity, and balance of stroke survivors [18, 19]. Within the myriad of possibilities with physical training, some researchers have proposed that resistance training (RT)—the kind of exercise that leads muscles to work or hold against an applied weight—may have a key role in the rehabilitation process after stroke as an effective strategy in the treatment of these individuals [20–22]. Much of the expectation around RT is due its capacity to modulate neuromuscular parameters (e.g., muscle mass, strength, and power) in samples composed of individuals who present a similar phenotype to the patient with a stroke, such as older adults who commonly show a nonconducive physiological environment due to neuromuscular denervation, low-grade inflammation, and functional incapacity [23, 24].

In this sense, an increasing number of evidence have been published in the last years demonstrating the benefits of RT in stroke patients. This phenomenon encourages authors to publish a review discussing the potential clinical variables that have not been evaluated in the context of exercise training and are extremely important to be measured in stroke survivors, as, for example, cognition, aphasia, and fatigue to quote a few [18]. Moreover, a recent systematic review with metanalytic regression suggests that most RT studies present a considerable number of biases, strongly limiting possible inferences about the data [19].

In addition to these concerns regarding the internal and external validity of these data, very little has been discussed about the design of the RT programs proposed in these studies. This kind of consideration seems to be important because the manipulation of the RT variables (rest interval between sets and exercises, number of sets, number of repetitions, intensity, duration of training, and weekly frequency) leads to different cardiovascular, metabolic, and neuromuscular responses [25–28]. Furthermore, many healthcare consumers—with or without previous scientific training in exercises sciences—base their clinical practices on exercise training studies. Lastly, it is noteworthy that the effectiveness or not of the RT program is dependent on the organization of its variables. Therefore, we indicate the limitations associated with RT prescription will collaborate with future investigations that aimed to understand the potential of RT to collaborate with rehabilitation in stroke survivors.

Thus, the current study aimed to discuss critically the main aspects regarding the RT prescription in programs with stroke patients, indicating the benefits of this type of training when all variables of importance are fully incorporated, as well as suggesting how studies may be better designed in an attempt to improve their internal and external validity.

## 2. Materials and Methods

### 2.1. Data Sources and Searches

Relevant studies were identified through computerized and manual searches. For data collection, PubMed, Google Scholar, and PEDro databases were systematically searched from 2003 until August 2017 (last 15 years). The following keywords were used for our search:* stroke, cerebrovascular accident, cerebral vascular accident, resistance exercise, and resistance training*. This review is written in accordance with Preferred Reporting Items for Systematic Reviews and Meta-Analyses (PRISMA) guidelines.

### 2.2. Study Selection

The PICO (population, intervention, control/comparison, and outcome variables) model was used for inclusion criteria. Studies were chosen for inclusion if they met the following 4 inclusion criteria: (A) humans of both genders, aged over 18 years with chronic stroke; (B) structured resistance exercise program; (C) randomized controlled trials; (D) health benefits.

The screening was performed by 2 independent reviewers. For each article, any discrepancy between the 2 reviewers was resolved by discussion. In the first screening stage (titles plus abstracts), studies were included when both reviewers agreed they were eligible for inclusion or if there was doubt about whether to exclude them. In the second screening stage (full text), studies were included when both reviewers felt they met all the inclusion criteria.

These reviewers documented the methodological quality of studies and extracted relevant data. The following quality criteria were documented: baseline comparison of groups, randomization, all assessed outcomes, and details of participants (i.e., age, gender, time after stroke, and comorbid conditions).

### 2.3. Data Extraction

The following characteristics were recorded for all articles: type of study, author, year of publication, participants (time after stroke, sample size, and age), benefits of RT, and structured resistance exercise programs. This procedure was performed by 2 reviewers: one collected the data and the second double-checked it.

## 3. Results and Discussion

A total of 478 articles were identified initially. In total, 12 peer-reviewed articles were included based on our inclusion criteria. The 12 studies enrolled 424 participants (middle-aged or elderly) with chronic stroke. The extracted study and population characteristics, protocols, and outcomes are shown in Tables 1 and 2.

### 3.1. Beneficial Effects of Different Designs of RT in Stroke Survivors

The variables investigated could be didactically divided into five large constructs as follows: physical capabilities (i.e., muscle endurance [VO2 max, 6 minutes' walk distance, and peak aerobic capacity], muscle strength and power, and balance), body composition (i.e., body mass index, muscle mass), cognition (general and executive functions [working memory, verbal fluency tasks, attention, and speed of information processing]), quality of life elements (e.g., anxiety, pain), and blood cardiovascular risk factors (i.e., fasting insulin, HOMA-IR, 2 h plasma glucose, total cholesterol, LDL cholesterol, and HDL cholesterol). All these variables were improved after resistance exercise programs in individuals with stroke.

Tables 1 and 2 depict data about participants, such as time after stroke, sample size and age, type of study, author's name, year of publication, evaluations of the investigated variables, benefits of RT, and structured resistance training programs (rest interval between sets and exercises, number of sets, number of repetitions, intensity, duration of training, and weekly frequency). Regarding RT methodology and protocols we found the following characteristics: number of exercises, 1 to 7; number of series, 2 to 4; intensity, low, moderate, and high; weekly frequency, 2 to 4 days; duration of the protocol, 5 to 12 weeks; number of repetitions, 6, 8, 10, 15, and 20; and rest interval between sets and exercises when shown, 2 and 3 minutes.

To our knowledge, this is the first review that critically evaluated RT programs used in stroke patients. This research is important to help to inform the healthcare consumers, mainly strength and conditioning professionals, about the need to control the variables of RT in stroke patients. In addition, the present study provides trainers and coaches with examples of well-controlled RT protocols and their benefits on the prognosis of stroke patients [29–33].

The main finding of this literature review lies in the fact that only 5 studies clearly laid out the main variables of RT used. The other 7 randomized controlled trials linking RT to stroke lack any description of rest interval between sets and exercises. According to some researchers, this important variable is widely used in studies involving resistance exercises [34–46]. In this context, we attempted to scrutinize how researchers dealt with the variables involved in RT and the associated benefits for poststroke patients.

Physical capabilities are extremely benefited after RT programs. Interestingly, the different RT designs might elicit different adaptations. Due to the principle of specificity—where it is postulated that a physical capacity is increased when the stimulus is similar to its performance (e.g., muscle strength is improved in response to resistance training)—most studies have focused on the capacity of RT to elicit significant improvements on the neuromuscular tract. This hypothesis has been confirmed, once numerous findings demonstrated that different designs of RT might cause improvements on dynamic, isokinetic, and isometric muscle strength, muscle power, and muscle tone of the nonparetic (muscle strength: +38.2%; muscle power: +28.5–61%) and paretic limbs (muscle strength: +31.4%; muscle power: +33.0–61%) of stroke survivors [31, 32, 47–51]. In fact, the studies differ in the number of repetitions (6–20), sets (2–4), sessions per week (2-3), intensities (low [50% 1RM], moderate [~70% 1RM], and moderate to high/high [80% 1RM]); pattern of muscle contraction (eccentric, concentric, or both), trained limb (paretic, nonparetic, or both).

In an elegant study performed by Lee et al. [52], for example, poststroke patients were submitted to a progressive resistance training program that consisted of two sets of eight repetitions at 50% of 1RM (in the first two weeks) and at 80% of 1RM in the following weeks. To ensure that the participants performed exercises in the target intensity, researchers monitored the perceived exertion through a Borg Perceived Exertion Scale. Results indicated that the progressive RT was able to significantly improve muscle strength, muscle power, and muscle endurance. Furthermore, authors observed that these results were not reached by volunteers that had performed a high-intensity cycling exercise (85% of VO2 peak). Nevertheless, as aforementioned, if a lower intensity was approached, as performed by Ouellette et al. [49], similar improvements in muscle capacity were observed.

It is important to mention that these improvements in muscle strength and power can be associated with elevated walking velocity and performance, balance, and transfer capacity (i.e., timed up and go [TUG]), as demonstrated by Fernandez-Gonzalo et al. [31], Flansbjer et al. [32], Severinsen et al. [48], and Ouellette et al. [49], as well as self-reported function [49]. These data have high external validity because stroke patients commonly show a high prevalence of motor impairment (67.0%), which is associated with poor outcomes, such as low quality of life and falls risk [53, 54], and predominantly characterized by low walking ability (88%), poor balance (51.4%), and dysfunction on mobility (54.1%). Moreover, improvements in self-reported function indicate that stroke patients are feeling better to perform the basic, instrumental, and advanced activities of daily living, probably suffering less with muscle fatigue and weakness.

On the other hand—different from muscle strength, which does not seem to be much influenced by different structures of RT—changes on aerobic capacity may be dependent on the organization of the RT variables. Indeed, Severinsen et al. [48] submitted stroke patients to a classical lower limbs RT program for 12 weeks. The prescription of exercise was based on three sets of 8 repetitions at ~50–80% of 1RM. The findings did not demonstrate significant alterations on aerobic capacity.

When poststroke survivors underwent a moderate to high/high-intensity RT based on three sets of 8 repetitions at 80% of 1RM [52], the authors observed not only an ameliorated muscle strength and power, but also an increased muscle endurance in both limbs—paretic and nonparetic—which was considered as the maximal number of repetitions that the volunteers could achieve in the 30s. Similarly, volunteers of Ivey et al. [47] showed significant improvements in peak oxygen consumption (+6%) and muscle endurance (paretic: +178%; nonparetic: +161%) after a RT program. However, the protocol was based on two sets of 20 repetitions until failure in three exercises for each lower limb member.

These data strongly highlight the importance of the right modulation of RT variables on organic adaptations, mainly the use of specificity in the context of muscle endurance. As stated by Lee et al. [52] daily activities frequently require the capacity to sustain muscle contraction for long periods. Interestingly, muscle endurance was improved after RT program with an elevated intensity (80%) [52], not in concordance with literature, because authors believe that programs with a high number of repetitions and a low intensity, such as that performed by Ivey et al. [47], are more probable to cause this type of adaptation due to biochemical responses. However, it is possible that the threshold to improve muscle endurance in patients with chronic diseases (e.g., stroke) is lower than in healthy patients. The plausibility of this theory is based on the findings that demonstrated increased aerobic capacity in patients with heart failure after RT [55].

The monitoring of morphological parameters is another important clinical aspect in stroke patients, once several evidences have been discussing that a sarcopenic phenotype—characterized by a decreased lean muscle mass followed by impaired muscle strength and/or functionality—is showed by these patients [5, 56]. In nonstroke patients, exercise intensity has a key role in the hypertrophic response to RT, so that most studies have demonstrated that RT programs at moderate to high and high intensities demonstrate a superior capacity to elicit improvements on muscle mass compared with RT programs with lower intensities [57].

In the context of stroke, just a few randomized clinical trials have investigated the impact of different RT programs on morphological parameters. In one of the few studies, Fernandez-Gonzalo et al. [31] decided to investigate the impact of a RT program with an overload on eccentric contractions in the cross-sectional area (CSA) of quadriceps muscle of stroke survivors. This approach is interesting because most interventions focus on muscle contractions composed of concentric and eccentric actions. On the other hand, eccentric muscle contractions have been used as a tool to induce muscle damage, for example, since during this kind of muscle contraction a lower number of fibers are working, leaving the muscle more susceptible to damage, collaborating to the study of delayed onset muscle soreness, inflammation, and hypertrophic response [58–61].

Nevertheless, some groups have proposed that RT programs composed of isolated eccentric contractions might propitiate superior gains in muscle mass and strength compared to concentric training programs [62]. In this sense, stroke survivors were submitted to a RT program based on eccentric overload in a flywheel characterized by 4 sets of 7 maximal knee extensions of the more-affected lower limb.

The findings demonstrated an increased CSA after 12 weeks of RT. This increase in lean muscle mass could help to explain the higher muscle strength and power, as well as walking speed observed in stroke patients undergoing an eccentric RT program in comparison with volunteers who performed a concentric RT program [50], so that taken together data demonstrate a potential superior effectiveness of eccentric RT programs in comparison with concentric RT programs.

However, unfortunately, these are isolated evidences and more studies are still necessary to understand the importance of this kind of muscle contraction in the morphological and neuromuscular parameters of stroke survivors. Furthermore, future studies should utilize protocols of eccentric overload RT training with higher external validity than the proposed one by Fernandez-Gonzalo et al. [31] and Clark and Patten [50] since flywheels and isokinetic equipment are expensive and limited to be used in the context of health promotion.

Regarding cognition, data have demonstrated that stroke patients show cognitive impairment rates range from 20% to 70%, depending on the type of diagnosis [63]. These data deserve concern because this phenomenon indicates that stroke survivors present a high risk to develop dementia [63]. Between the several domains that compose cognition, executive function, which is an essential cognitive domain to maintenance of autonomy and independence during aging [64], seems to be one of the most affected domains after the cerebrovascular event, since evidence suggests that stroke survivors demonstrate a faster decline in this cognitive domain (0.63 points/year) [65].

Intriguingly, even in the face of such data, a recent review indicated that studies are not investigating the impact of RT programs on cognitive parameters of poststroke survivors [18]. In fact, to the best of our knowledge, just one study aimed to investigate this phenomenon. In the trial of Fernandez-Gonzalo et al. [31], an improved executive function (working memory, verbal fluency tasks, attention, and speed of information processing) were observed after the RT program. However, as aforementioned, this study was performed on a specific machine and most studies are still necessary.

After the cerebrovascular event, stroke patients present an increased prevalence of cardiometabolic disorders, leading to an elevated risk of recurrent stroke or other cardiovascular events (e.g., myocardial infarction). In this sense, Zou et al. [66] proposed a RT program based on three sets of 15 repetitions until muscle failure of the leg press, leg extension, and leg curl. This kind of RT training might be beneficial because of two factors: (a) a larger number of repetitions may elicit a larger translocation of glucose transporter 4 (GLUT4) to sarcolemma and, consequently, larger glucose uptake; (b) muscle contractions until failure might elicit a hypertrophic muscular signalization, inducing increase in muscle mass [57], the largest site of glucose uptake in the human organic system. Data demonstrated that several blood cardiovascular risk factors (i.e., fasting insulin, HOMA-IR, 2 h plasma glucose, total, LDL, and HDL cholesterol) were improved after the RT program, regardless of the alterations on body mass.

### 3.2. What Were the Main Limitations Observed in RT Prescriptions?

The acute and chronic organic responses to RT are dependent on the organization of its variables (rest interval between sets and exercises, number of sets, number of repetitions, intensity, duration of training, and weekly frequency). Regarding the acute effects, patients with and without hypertension, for example, experienced postexercise hypotension (PEH) in RT protocols performed at moderate intensity [67–69]. However, an increasing number of evidences have indicated that exercise cadence might have a key role in this phenomenon [70]. In turn, chronic changes on cognitive parameters do not seem to be intensity-dependent, but time-dependent [71–75]. On the other hand, muscle strength is increased after RT protocols composed of elevated tension [25].

Regardless of its beneficial effects, for a long time, RT was not recommended for patients clinically diagnosed with any cardiovascular diseases [76]. This conduct was assumed because some experiments demonstrated an important increase in blood pressure values during the performance of RT [77]. However, recently, numerous evidences have to indicate that RT may be carried out safely if the variables are controlled [78].

This brief explanation demonstrates the importance of organizing the RT variables for effectiveness and safeness of the exercise program. Stroke survivors are, undoubtedly, patients who deserve special attention during the performance of the exercise training, once they present increased risk of falls, muscle weakness, exacerbated fatigue, autonomic dysfunction, and hemodynamic instability, to quote a few clinical symptoms. Therefore, researchers should perform a detailed explanation about the RT protocols in an attempt to provide the information necessary for healthcare consumers to replicate—within their means—the protocols in their clinical practice. Unfortunately, despite the abovementioned beneficial effects, most studies have failed to detailedly describe the organization of the RT variables. The main limitations include an insufficient or even absent description of the rest intervals between sets and exercises, the name of exercises, the cadence of muscle contraction, and the tools used for RT prescription.

Regarding the description of rest intervals between sets and exercises, this variable was not described in almost all investigations. This is curious because in our clinical practice, as well as in the laboratory, this issue has been extensively discussed because an unsatisfactory rest interval may negatively interfere with expected benefits. As Fleck [37], Kraemer et al. [40], and Ratamess et al. [42] have pointed out, cardiorespiratory and metabolic responses are affected by the duration of intervals of rest between exercises and sets. In addition, rest intervals have a key role in the neuromuscular adaptations as stated by Robinson et al. [44], who demonstrated that greater rest intervals were associated with greater strength gain when compared to shorter intervals.

In the RT protocols of Ouellette et al. [49] and Lee et al. [51, 52], for example, researchers failed to describe the rest intervals among the sets and exercises. However, authors reported that RT sessions were performed at 70–80% of 1RM, which may elicit accumulation of metabolites. This phenomenon might be a problem in the context of stroke, because patients present exacerbated fatigue and muscle weakness, impairing the clearance of metabolic debris. Therefore, it is possible to infer that authors used ≥2 minutes of interval among the series, as recommended for this kind of RT [79]. However, a long interval may not be enough to elicit the necessary stress to cause muscular adaptations. Consequently, the replicability of these studies would be compromised.

Another meaningful aspect is the description of the exercises that were used in the RT program. This occurs because hemiparesis markedly impairs the pattern of movement and, in the clinical practice, the use of specific exercises is not always possible. In fact, we have observed that several adaptations should be performed in the pattern of movement/exercise in order to contemplate the muscular action that can be performed by the patients.

If the purpose of the session of RT was to exercise the pectoralis major, for example, it is possible to infer that researchers had to adapt the exercise to contemplate the muscle group, since stroke patients present several limitations that may make it impossible to conduct the exercise in the machines. Thus, future studies should describe the name of the exercises that were used in the experiments, as well as indicating if any kind of adaptations was performed.

The method used for prescription of RT intensity has been widely discussed not only in the context of* exercise as medicine*, but also in the sports science field. This occurs because evidence suggests that results on 1RM test for lower limb may variate until 14% of a series of nonsuccessive tests [80].

Curiously, Severinsen et al. [48] reported problems to work with the 1RM method, so that researchers realized that values could be under- and/or overestimated, making the inferences about the zone of intensity difficult where individuals have performed the RT program. Alternatively, Lee et al. [51] in addition to 1RM used a Borg Perceived Exertion Scale, which has been indicated as a valuable tool to exercise prescription [81, 82]. Indeed, RPE allows the adjustment of the load in each session of exercise, differently from the 1RM method, which needs a new battery of evaluations.

However, most researchers have discussed that a previous period of adaptation is necessary before the use of subjective scales. This deserves to be reinforced in patients with stroke because a longer period might be necessary for them since, as aforementioned, they commonly present cognitive impairment. Nevertheless, this issue still must to be investigated.

Lastly, it is noteworthy that some concepts have been wrongly stated. This may lead to wrong conclusions and prescriptions between healthcare consumers. In some studies [49], authors declared that a progressive RT was performed. However, RT intensity was maintained during the whole period. It is important to clarify that a progressive RT is not based on the adjustments on load due to muscular adaptation, but a progressive increase in load—from 50% to 80%, for example—is necessary. This kind of RT was demonstrated in the study by Lee et al. [52]. Furthermore, some authors declared that the RT program was performed at high intensity when the load was around 70% of 1RM. Inferences about high-intensity RT must be made carefully based on these data because most authors declare that training loads at 70% of 1 RM correspond to moderate intensity [83, 84].

**Figure 1:**
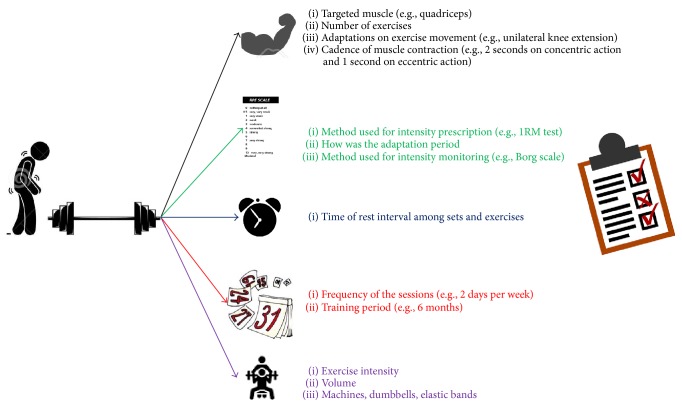
Minimal amount of information that should be described in experiments that use RT programs.

Figure 1 shows the minimal amount of information that should be described in experiments that use RT programs.

## 4. Conclusions

After analyzing the 12 randomized controlled trials, we found that only 5 studies may indeed help health and physical conditioning professionals in exercise prescription for stroke patients, since they did detail and control the main RT variables.

More randomized controlled trials with tight control of the main variables of the RT should be undertaken so that health professionals may be more scientifically informed when prescribing resistance exercises for individuals with stroke. Individuals with stroke may be considered patients at risk; therefore, it is critical to be cautious in exercise prescription. The findings of the present study may further inform health professionals on the importance and necessity of using the main variables (rest interval between sets and exercises, number of sets, number of repetitions, intensity, duration of training, and weekly frequency) in the search for benefits for individuals with stroke.

## Figures and Tables

**Table 1:** Benefits and description of different RT programs in individuals with stroke (*n* = 5); control of main variables.

Authors	Samples	Protocols	Assessments	Results
Aidar et al. (2012)	24 subjects, mean age of 51,7 ± 8 with chronic stroke (≥6 months) (15 men and 9 woman), were randomized to either the Experimental Group (EG) or Control Group (CG)	Control of main variables of resistance training (rest interval between sets and exercises, number of sets, number of repetitions, intensity, duration of training, and weekly frequency).	State-Trait Anxiety Inventory (STAI)	↓ state anxiety of the EG when compared to CG

Aidar et al. (2016)	24 subjects, mean age of 51,7 ± 8 with chronic stroke (≥6 months) (15 men and 9 woman), were randomized to either the Experimental Group (EG) or Control Group (CG)	Control of main variables of resistance training (rest interval between sets and exercises, number of sets, number of repetitions, intensity, duration of training, and weekly frequency).	Quality of life	↑ quality of life of the EG when compared to CG

Fernandez-Gonzalo et al. (2016)	32 subjects with chronic stroke (≥6 months) were randomized to either the training group (TG), age (years) 61.2 ± 9.8, or Control Group (CG) age (years) 65.7 ± 12.7	Control of main variables of resistance training (rest interval between sets and exercises, number of sets, number of repetitions, intensity, duration of training, and weekly frequency).	Skeletal muscle size, strength and power, functional performance, and cognitive function	↑ quadriceps volume of the more-affected leg, ↑ muscle power, ↑ balance, ↑ gait performance tasks, ↑ attention and speed of information processing in TG; CG showed no changes

Flansbjer et al. (2008)	24 subjects, mean age 61 ± 5 (14 men and 10 woman) with chronic stroke (≥6 months), were randomized to either the training group (TG) or Control Group (CG)	Control of main variables of resistance training (rest interval between sets and exercises, number of sets, number of repetitions, intensity, duration of training, and weekly frequency).	Muscle strength, muscle tone, timed “Up and Go,” fast gait speed and 6-minute walk tests, and perceived participation by stroke Impact scale; all measurements were made before and after the intervention and at follow-up 5 months after the intervention	↑ muscle strength, timed “Up and Go,” and perceived participation of the TG group when compared to CG

Flansbjer et al. (2012)	18 subjects, mean age 66 ± 4 with stroke, were randomized to either the training group (TG) or Control Group (CG)	Control of main variables of resistance training (rest interval between sets and exercises, number of sets, number of repetitions, intensity, duration of training, and weekly frequency).	Muscle strength, muscle tone, timed “Up and Go,” fast gait speed and 6 minutes' walk tests, and perceived participation by stroke Impact scale; the assessments were repeated at the 4-year follow-up	↑ muscle strength of the TG group when compared to CG

**Table 2:** Benefits and description of different resistance training programs in individuals with stroke (*n* = 7); lack of description of some variable.

Authors	Samples	Protocols	Assessments	Results
Clark and Patten 2013	35 subjects with chronic stroke (≥6 months) were randomized to either the concentric resistance training group (CON), age (years) 59.7 ± 10.9, or an eccentric resistance training group (ECC), age (years) 63.2 ± 10.6	Lack of description of rest interval between sets and exercises	Walking speed, assessment of and neuromuscular activation and power	↑ bilateral neuromuscular activation, ↑ walking speed of the ECC group when compared to CON

Ivey et al. 2017	30 subjects (21 men and 9 woman) with chronic hemiparesis (>6 months poststroke) were randomized to either the Strength Training (ST), age (years) 57 ± 14, or Stretch Control (SC), age (years) 55 ± 9	Lack of description of rest interval between sets and exercises	Skeletal muscle endurance, one-repetition maximum strength, 6 minutes' walk test, 10-meter walk speeds, and peak aerobic capacity	↑ skeletal muscle endurance, ↑ 6 minutes' walk test, ↑ peak aerobic capacity of the ST group when compared to SC group

Lee et al. 2008	48 subjects mean age of 63 ± 9 with stroke (≥3 months) were randomized to either the control or progressive resistance training (PRT) or cycle or combined	Lack of description of rest interval between sets and exercises	6 minutes' walk test, habitual and fast gait velocities, stair climbing power, cardiorespiratory fitness, muscle strength, power, and endurance and psychosocial attributes	↑ muscle strength, ↑ power, ↑ endurance, ↑ cycling peak power output; ↑ self-efficacy to PRT

Lee et al. 2010	48 subjects, mean age of 63 ± 9 with stroke (≥3 months), were randomized to either the progressive resistance training (PRT) + cycling or PRT + sham cycling or sham PRT + cycling or sham PRT + sham cycling	Lack of description of rest interval between sets and exercises	Muscle strength, peak power, muscle endurance	↑ power limb muscle strength, ↑ peak power, ↑ muscle endurance the PRT + sham cycling group when compared to sham PRT + cycling or sham PRT+ sham cycling groups

Ouellette et al. 2004	42 subjects with stroke (≥6 months) were randomized to either the progressive resistance training (PRT) group, age (years) 65.8 ± 2.5, or Control Group (CG), age (years) 66.1 ± 2.1	Lack of description of rest interval between sets and exercises	Muscle strength, functional performance, Late Life Function and Disability Instrument (LLFDI)	↑ muscle strength, ↑ self-reported function. and disability of the PRT group when compared to CG

Severinsen et al. 2014	48 men with chronic stroke (≥6 months) were randomized to the Aerobic Training (AT) group, age (years) 69, resistance training (RT) group, age (years) 68, and Sham Training (ST) group, age (years) 66	Lack of description of rest interval between sets and exercises	Muscle strength, peak aerobic capacity, 6 minutes' walk test, fast 10 m walking speed	When compared to groups AT, RT, and ST important effects were not observed on walking velocity or walking distance; ↑ muscle strength, ↑ walking velocity for RT group

Zou et al. 2015	51 subjects with chronic stroke (≥6 months) were randomized to either the Experimental Group (EG), age (years) 52.3 ± 6.9, or Control Group (CG), age (years) 51.4 ± 7.2	Lack of description of rest interval between sets and exercises	Blood glucose level, serum lipids profiles, body mass index, muscle strength	↓ fasting insulin, ↓ HOMA-IR, ↓ 2 h plasma glucose, ↓ total cholesterol, ↓ LDL cholesterol, ↑ HDL cholesterol, ↑ muscle strength of the EG when compared to CG

## Abbreviations

1RM: 1-Repetition Maximum
ACSM: American College of Sports and Medicine
AHA: American Heart Association
CSA: Cross-sectional area
GLUT4: Glucose transporter 4
HDL: High density lipoprotein
HOMA-IR: Homeostatic model assessment-insulin resistance
LDL: Low density lipoprotein
PEDro: Physiotherapy Evidence Database
PEH: Postexercise hypotension
PICO: Population, intervention, control/comparison, and outcome variables
PRISMA: Preferred Reporting Items for Systematic Reviews and Meta-Analyses
RT: Resistance training
TUG: Timed “Up and Go.”
